# Prevalence of Varied Coat Coloration in a Yellow-Throated Marten (*Martes flavigula*) Population

**DOI:** 10.3390/ani11102838

**Published:** 2021-09-29

**Authors:** Yinan Gong, Guojing Zhao, Huixin Yang, Yan Li, Mengyu Tan, Ning Wang, Jianping Ge, Haitao Yang, Limin Feng

**Affiliations:** Ministry of Education Key Laboratory for Biodiversity Science and Engineering, National Forestry and Grassland Administration Key Laboratory for Conservation Ecology of Northeast Tiger and Leopard National Park, Northeast Tiger and Leopard Biodiversity National Observation and Research Station, National Forestry and Grassland Administration Amur Tiger and Amur Leopard Monitoring and Research Center, College of Life Sciences, Beijing Normal University, Beijing 100875, China; gongyinan07@163.com (Y.G.); 15754501939@163.com (G.Z.); cyn@mail.bnu.edu.cn (H.Y.); lan02130420@163.com (Y.L.); tmy196103@163.com (M.T.); wangning@bnu.edu.cn (N.W.); gejp@bnu.edu.cn (J.G.)

**Keywords:** abnormal coloration, camera trap, *Martes flavigula*, yellow-throated marten, Northeast China

## Abstract

**Simple Summary:**

Abnormal coloration is very rare in any given population of wildlife; however, our research identified a yellow-throated marten population with a high ratio of this phenomenon for the first time. Across the main distribution of the species with relevant observational data, we observed abnormally-colored martens in only Northeast Tiger and Leopard National Park. Abnormal coloration had a variety of forms and individuals with white paws that accounted for a larger proportion of the overall population than normal individuals. This shows heritable variation in the region, which is worthy of further research.

**Abstract:**

Mammalian coat color is determined by heritable variations such as disease, nutrition, and hormone levels. Variation in animal coat color is also considered an environmental indicator and provides clues for the study of population genetics and biogeography. Records of abnormal coloration in the wild are rare, not only because it is often selected against, but also because of the difficulties in detection of the phenomenon. We used long-term camera-trapping data to first report abnormal coat coloration in yellow-throated marten (*Martes flavigula*) in China. Six types of abnormal coloration were found only in the Northeast Tiger and Leopard National Park, Northeast China, which were not reported in other regions in China. A total of 268 videos of *Martes flavigula* contained normal coloration, 455 videos of individuals of the species contained abnormal coloration, 437 contained the ‘gloving’ type (martens with de-pigmented front toes, paws or lower forelimbs), while the remaining other 18 videos contained five types (different degrees of white-spotting and dilution). The higher relative abundance index (0.428, ‘gloving’ to 0.329, normal) and wide distribution area of the ‘gloving’ type indicated that this abnormal coat coloration type is usual in Northeast China, which may reflect genetic variability in the local population. These records will contribute to further research on animal coat color and its corresponding adaptive strategy.

## 1. Introduction

Mammalian coat color is often determined by heritable variables or other factors, such as seasonal coloration, related with hormones [1,2,3]; aging, leading to the change of melanin [4,5]; and disease, injury or nutrition-related phenomena [6,7]. Coloration differences may be driven by background matching, signaling, and physiological influences [8,9]. Although it is considered a plastic morphological characteristic [10], coat color has certain reference significance in traditional taxonomy [11,12], reflecting polymorphisms and biodiversity. Abnormal coloration is common in domestic animals, especially selectively bred animals, but it is rare in populations of wildlife because abnormal coloration is often selected against and considered a disadvantage [13,14,15]. Additionally, variation in animal coat color is sometimes considered an environmental indicator and can provide clues for studies on population genetics and biogeography [14,16]; frequent abnormal coloration in a population may reflect inbreeding [17] or a sharp decrease in history, causing founder effects [18,19,20,21,22].

Among captive animals, rodents [23], dogs and cats [24,25,26], and horses [27,28] are relatively well studied. Among wild animals over the world, abnormal (anomalous) coloration is more recorded in birds [7,15]. In mammals, Cetartiodactyla (cetaceans), Eulipotyphla, Chiropter, Carnivora, and Rodentia [15,18] account for a considerable proportion. Among them, some marine or subterranean animals and bats rely less on vision, and rodents are under intensive predation [18]. The large number of records of abnormal coloration within the family Mustelidae may be related to their niche as mesocarnivores. Within the family Mustelidae, in previous studies, abnormal coloration has been observed sporadically in wild-type tayras (*Eira barbara*) [29,30], Neotropical otters (*Lontra longicaudis annectens*) [31], Eurasian otters (*Lutra lutra*) [32], oriental small-clawed otters (*Aonyx cinereus*) [33], fishers (*Pekania pennanti*) [15], and Eurasian badgers (*Meles meles*) [34]. Whitish facial markings have also been observed in long-tailed weasels (*Mustela frenata*) in Central America, Mexico, and the southern USA [35]. Research on the coloration of the American mink is rather thorough and is mostly to the benefit of the fur market [36].

Mammalian coat color results from the presence of melanin, which exists in two different forms: eumelanin, responsible for black and brown coloration, and pheomelanin, responsible for red and yellow coloration [37]. In mammals, according to previously published studies, abnormal coloration involving hypopigmentation can be divided into several phenotypes, including but not limited to the following:

(i) Albinism, characterized by a total lack of melanin, is caused by the absence of tyrosinase, leading to de-pigmented skin, fur, and eyes [23,38,39,40].

(ii) Leucism, caused by a partial or total lack of pigmentation throughout the whole body, but with normal coloration maintained in the eyes [18,38]; some publications have reported that ‘leucism’ rarely affects hairless body parts, such as the nose and feet, and never affects the iris of the eye [31,41,42,43], leading to de-pigmented fur only.

(iii) Piebaldism, although it only refers to white patches determined by piebald gene loci, is always used to refer to all white spotting [23,38]; this spotting occurs when melanin is absent from some or all of the areas in which it is normally present and is sometimes classified as partial leucism. Variants in different genes cause spotting patterns ranging from sparse white markings to total body discoloration; some piebald phenotypes are accompanied by hypopigmented eyes [27,28]. We applied the term ‘white spotted’ [7,26,44] to these phenotypes in our study. Animals with de-pigmented front toes, paws, or lower forelimbs (‘gloving’, according to cat fanciers [24,26,45]) are extremely rare in the wild and are usually observed in selectively bred domestic animals [25].

(iv) Dilution, characterized by paler and more silvery coloration than normal [23]. Hypomelanism refers to insufficiently pigmented skin and has been applied by Davis [46] to all different types of dilutions [7].

Records of abnormal coloration in mammals are often misclassified [38] due to difficulties in capturing specimens and performing genetic testing. Moreover, different terms are used differently by zoologists and pigmentation/genetic scientists; for instance, ‘partial albino’, which has been demonstrated to not exist, continues to be applied [7]. Moreover, it is difficult to describe all mammals with a simple standard because of the substantial variation in geno-/phenotypes.

The yellow-throated marten, a small diurnal carnivore with a wide prey range, usually travels and hunts in pairs or sometimes packs. Yellow-throated martens maintain relatively large ranges that are actively patrolled [47]. Depigmentation diversity in this species has never been officially documented. However, coat color is an important indicator for the taxonomy of the subspecies of yellow-throated martens; those which inhabit the Malay Peninsula and South Burma (*M. fpeninsularis*) and Hainan Island, China (*M. fhainana*) have dark stripes behind the ears, while the coat color of martens in Northeast Asia is lighter [11,48]. Related research on the yellow-throated martens remains very scarce. Meanwhile, abnormal coloration at a population level [17,49] is difficult to study for the lack of data from systematically long-term monitoring [18,50], and the understanding of location and frequency of the phenomena is important [13]. Here, the data of millions of videos and pictures from long-term camera trap surveys were analyzed to: (1) find all abnormal coloration types that occur in the population, in order to reveal depigmentation diversity in this species; (2) preliminarily estimate the abundance and distribution of abnormally colored yellow-throated martens. Because of the lack of acknowledged terms of mammalian coat color classification, the descriptive term ‘white spotted’ is applied here to describe the martens with different degrees of white patches. ‘Gloving’ describes those with depigmentation restricted to front toes, paws or lower forelimbs, and ‘abnormal coloration’ [7,15] includes all of the above.

## 2. Materials and Methods

### 2.1. Data Collection

**Figure 1:**
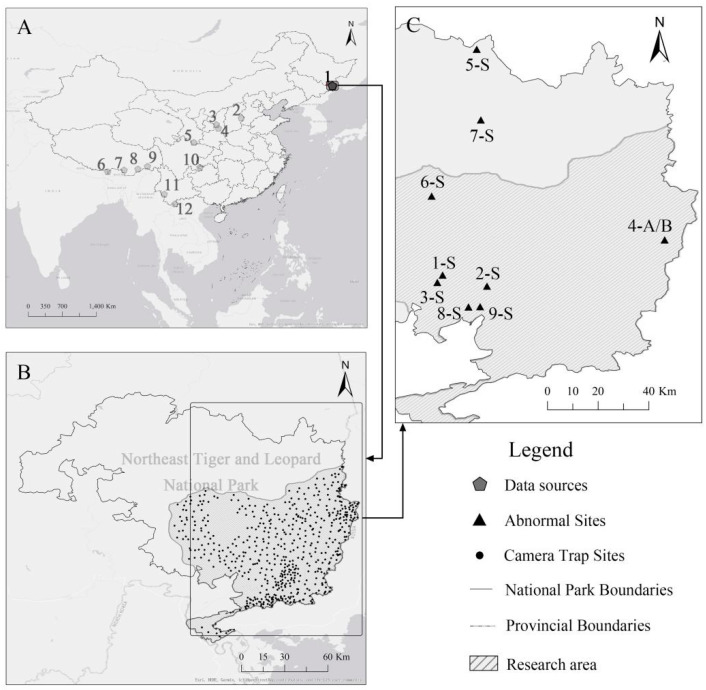
(**A**) Areas with positive results for yellow-throated marten observations. 1: Northeast Tiger and Leopard National Park; 2: Jinzhong; 3: Ziwuling; 4: Qiaoshan; 5: Giant Panda National Park; 6: Yadong; 7: Cona; 8: Medog; 9: Zayu; 10: Xishui; 11: Nangunhe; and 12: Xishuangbanna. (**B**) Distribution of all 597 working cameras, from 1 January 2014 to 31 December 2015. (**C**) Locations where 18 abnormally-colored (except ‘gloving’) records were obtained in Northeast Tiger and Leopard National Park.

Long-term camera trap survey networks were established across 7 provinces in China starting in 2007 (Figure 1A), each covering over 1000 km^2^, and provided considerable records of yellow-throated martens. Studies that report the home range of yellow-throated martens in Northeast Asia are scarce. However, in Thailand, they showed an annual range of 7.2 km^2^ (±4.3) and traveled 966 m/day (±834) on average [47]. Likewise, pine marten (*Martes martes* L.) [51] and American marten (*Martes americana*) [52] showed close results. Therefore, our research areas are large enough to cover home ranges of sufficiently large number of individuals. Camera traps were placed along trails, roads, and ridges, which are natural routes for tigers, leopards, and other wildlife, 0.5–5 km apart and were operated continuously throughout the year. No bait was applied to attract the animals.

The camera trap survey network in Northeast Tiger and Leopard National Park [53,54,55] has been operating consecutively for 14 years. In this study, we collected data of yellow-throated martens starting in 2007 to find abnormally-colored martens. Continuous observation data from 597 camera trapping locations (Figure 1B) over the course of 2 years (from 2014 to 2015) were analyzed to understand the distribution and abundance of martens with color variation (‘gloving’).

### 2.2. Records Processing and Analysis

We analyzed the ‘gloving’ type separately from the white spotted and dilute types because of its frequent occurrence, and due to the difficulties in individual identification, the relative abundance index (RAI), which is widely used in species that cannot be individually identified [56,57,58], was calculated to analyze this phenomenon. RAI, which was based on independent events from 1 January 2014 to 31 December 2015 in the research area, was applied to measure the relative abundance of normal and ‘gloving’ yellow-throated martens; independent events and the RAIs of normal, ‘gloving’, unknown (those from which we could not see the paws) and all yellow-throated martens (types not distinguished, aiming to study the distribution of the whole population) were calculated separately.

Independent events at each camera trapping location are defined as: (1) records of different individuals or species; (2) records of individuals of the same species taken at least 0.5 h apart (when individuals cannot be identified); and (3) discontinuous records of the same individual. A video meeting any of these criteria was considered an independent event. In this research, records of the same type in 1 day are counted as one independent event in order to better avoid overcounting. RAI is the estimated number of independent events acquired per 100 trap-days [57], calculated as follows: RAI = 100 × ∑*_i_*_=1_*N_i_*/∑*_i_*_=1_*Trapday_i_*, where *i* is camera trap location and *N_i_* is the number of independent events at the *i*th location.

The RAI was also applied for relative abundance mapping in ArcGIS (v10.7). Spatial interpolation (SI) is widely used in geographical research and is applied to estimate relative abundance and other indices through simulation [59,60]. Kriging is a commonly used spatial interpolation method to estimate relative abundance indices; it estimates the value of unknown points by using the weights and values at known points [61,62], and was applied by ArcGIS [63] in this study to estimate the potential distribution of yellow-throated martens.

## 3. Results

### 3.1. White Spotted and Dilute Marten Observations

**Figure 2:**
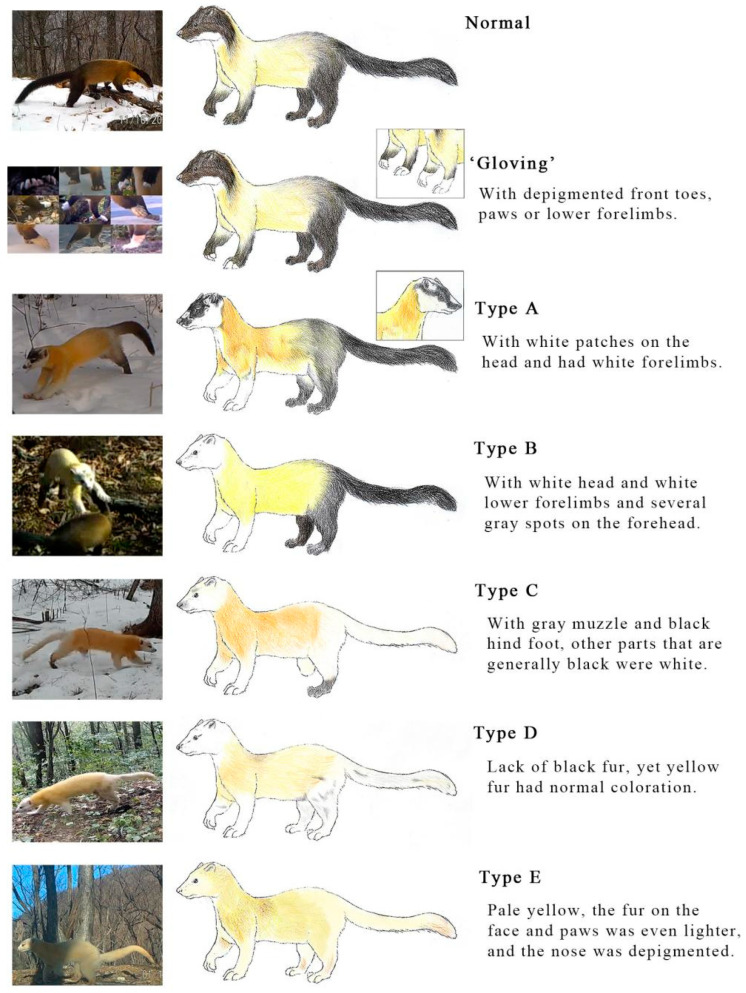
Hand painting and camera trap records of abnormally-colored yellow-throated martens in Northeast Tiger and Leopard National Park, Northeast China. Type C is a mirror painting.

The 18 video clips of white spotted and dilute martens (Table 1, Figure 1C and Figure 2) from all 455 abnormally colored yellow-throated martens are described as: Type A, white patches on the head and white forelimbs; Type B, white coloration on the head and the lower forelimbs and several gray spots on the forehead (the trunk of the body was bright yellow, and the nose was normally colored); Type C, gray on the muzzle and part of the forehead, only the right hind foot was black (the left one was broken); other body parts that are generally black were white, and the yellow part of the body was normally colored; Type D, lack of black fur, yellow fur had normal coloration; and Type E, pale yellow, the color of the face and paws was even lighter, and the nose was pink (de-pigmented). We described Types A to D as ‘white spotted’ and Type E as ‘diluted/leucistic’. All individuals clearly experienced eumelanin loss.

### 3.2. High Proportion of ‘Gloving’ Records

**Figure 3:**
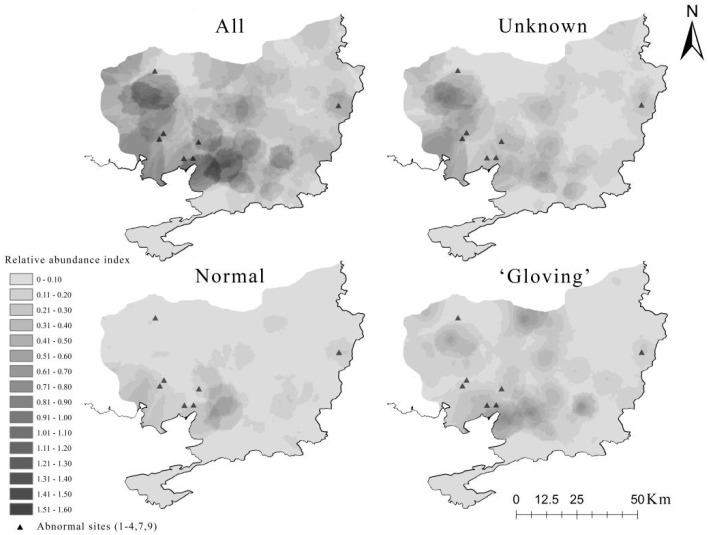
Relative abundance map of all yellow-throated martens (unknown, normal or ‘gloving’ not differentiated), and those with unknown, normal and ‘gloving’ during 2014–2015 in the research area in Northeast Tiger and Leopard National Park, China. Camera observations of white spotted and dilute martens coincide with the ‘gloving’ population distribution; abnormal sites 5 and 8 are not in the area.

From 2014 to 2015, we found that the ‘gloving’ martens outstripped normal martens in abundance (using RAI, Table 2) and distribution area (using Kriging, Figure 3). The distribution map of ‘gloved’ martens indicates that a large and stable population with this phenotype is widely distributed in the eastern part of the park (Figure 3). None of the specimens showed clear signs of pheomelanin disorder, while they all showed different degrees of eumelanin loss.

## 4. Discussion

Records of abnormal coloration in yellow-throated martens are valuable, as they indicate more generally natural coat color polymorphisms in mammals, providing a valuable opportunity to better understand mammalian coat color development and adaptive pigmentation in the future. Our records extensively supplement existing abnormal Mustelidae coloration records [15].

Chromatic disorders such as albinism and amelanism are said to be more common in tayras than in other mustelids [7], yet coat color-related research in Mustelidae is scarce, and the function is not well known. The diversity of coat color of yellow-throated martens may be a neutral mutation; long-lasting snow cover and dry leaves in Northeast China may provide concealment [15,64] for white spotted and dilute martens. Genetic alterations cause the majority of ‘gloving’, and the accumulation of such genetic alterations may explain the cause of depigmentation in Types A to E (camera observations of these types coincide with the ‘gloving’ population distribution, Figure 3).

Studies on such inherited characteristics can support future research on study populations under inbreeding or environmental stress [65]. It is known that abnormal coloration may have negative effects, such as increased visibility to prey, an increased risk of predation, and potential intraspecific exclusion [13,64,66]. According to fecal DNA analysis, top predators have not been found to prey on yellow-throated martens [55], and potential ecological risks may be reduced by adjustments along niche dimensions [67,68,69]. Research in northern China showed that yellow-throated martens have a relatively low spatiotemporal overlap with other mesocarnivores and spatially avoid apex predators [57]. Their wide range of prey may relieve challenges induced by a decreased efficiency in hunting. Additionally, no evidence has shown that low- to medium-grade spotting results in exclusion by groups, and their gregarious behavior may improve survival. Research on anomalous mesocarnivores may improve the understanding of populations containing these individuals, which may play an important role in ecosystem function, structure, or dynamics [70].

These mutations, compared to other random mutations causing depigmentation in the wild, are more stable and specific and usually occur in selectively bred or inbred domestic animals [24,25,44]. It is quite possible that the frequent occurrence of abnormally colored martens is also due to inbreeding among the small population in an isolated habitat (isolated population). Biogeographic analyses of *Martes flavigula* populations show that the species diversified into northeastern Asia from southern China long ago [71], and ‘gloving’ has been previously recorded rarely in northeastern Asia before, including the Russian Far East and South Korea (e.g., two images in two articles for instance) [72,73], indicating that the rare variation in the coat color of the species may originate in the region.

Causes of different or abnormal coloration vary. Ambiguous terms and those with unwarranted interpretations of the observed coloration should be avoided [7,74]. With increasing research and study, some existing terms have been replaced or clearly defined. Although it is not necessary to replace existing terminologies with modified ones [7], mechanisms of the phenomena should be considered. We recommend that terms should be used with caution, and reports based simply on observations (witness) should focus on descriptions that can be applied in later research; if uncertainty exists, specific explanations of mechanisms should be avoided.

Research on wildlife coat color variation is relatively difficult, nevertheless, it covers several contents, including recording (cases reports), behavior/ecological variables (evolutionary), and cellular mechanisms/morphology (developmental) [9]. Research on abnormally-colored tigers (*Panthera tigris*) (natural polymorphism originated from the wild, captive-maintained) focused on genetic information [75,76], and was not concerned with the influence of coat color variation on their survival and reproduction in wild populations. The inadequacy of monitoring methods and declining wild populations lead to the lack of field data about abnormally-colored wildlife, which makes the research of wildlife coat color variation extremely difficult. Benefiting from the extensive application of noninvasive camera trapping [34], research is promoted on the impacts of coat color variation on health, survival rate, behavior, and its role in population and evolutionary ecology in wildlife [15]. In this study, the generality that abnormal coloration (not albinism) has a significant adverse impact on health, survival, and reproduction may be doubted [34,77], because abnormally-colored yellow-throated martens are dominant in the north-east population and widely distributed in our study area. However, our research is only a start: First, we should unify the concept of the phenotype descriptions [7,38]; second, more attention needs to be paid to the combination of macro and molecular methods to explore the impacts of coat color variation on mechanism [9,78], which will promote future research on wildlife coat color variation.

## 5. Conclusions

In this study, we used the rare data of varied coat coloration in a yellow-throated marten population based on long-term camera trapping, to provide a starting point for further studies about the phenomena in wildlife. The data from camera trap makes it possible for researchers to accurately record and describe specimens/events with minimum interference and more comprehensive cognition. Beside the camera trapping data, it is still crucial to confirm the genetic causes from a molecular level about the abnormal coat coloration in wildlife. Thus, studies on a larger scale shall be carried on to define the range of the phenomena, to analyze ecological factors and inter-specific pressures, and to better understand the driven force of coat color variation from a macro to molecular level, to further understand the role of coat coloration in wildlife adaptive strategy.

## Figures and Tables

**Table 1:** Information about the 18 video records. Camera sites 1~9 contained nine different observation sites; ‘4-A/B’ refers to two cameras in a pair; ‘S’ refers to a single camera at the site. From 18 September~14 October 2012, nine videos of five independent events were captured. Type A, white patches on the head and white forelimbs; Type B, white head and lower forelimbs; Type C, gray on the muzzle and part of the forehead, and only the hind foot was black; Type D, lack of black fur; and Type E, pale yellow on the whole body, the color of the face and paws was even lighter, and the nose was pink (de-pigmented).

Records	Type	Camera Site	Date
R1	A	1-S	11 January 2015
R2	A	2-S	26 October 2016
R3	A	3-S	21 November 2016
R4	A	1-S	28 December 2016
R5~R13	B	4-A/B	18 September~14 October 2012
R14	C	5-S	15 February 2020
R15	D	6-S	28 August 2020
R16	D	7-S	26 September 2020
R17	E	8-S	13 January 2018
R18	E	9-S	18 January 2018

**Table 2:** Comparison of different types of martens from 1 January 2014 to 31 December 2015.

	Number of Observation Cameras	Percent of Observation Cameras	Number of Videos	Number of Independent Events	RAI
white spotted and dilute	9	/	18	14	/
Gloving	180	30.15%	437	381	0.428
Normal	143	23.95%	268	240	0.329
Unknown	245	41.04%	585	529	0.417
All yellow-throated martens (types not differentiated)	332	55.61%	1177	1021	0.909

Note: ‘Percent of observation cameras’ = Number of camera sites that captured footage of abnormally-colored martens/597 working cameras. Independent events of each type were calculated separately. ‘Unknown’ means the paws cannot be observed.

## Data Availability

The data presented in this study are available on request from the corresponding author.
